# Biogenesis, functional roles, and pathological implications of migrasomes

**DOI:** 10.1038/s41419-025-07943-z

**Published:** 2025-08-19

**Authors:** Xuexue Liu, Dun Liu, Heng Zhao, Kangxue Wu, Shaoping Yin, Xian Yang, Chuanqin Chen, Xiaolong Ma, Yonghuan Mao, Haixia Zhang, Lihua Shao, Siliang Wang, Xiao Du

**Affiliations:** 1 Department of Pharmacy, Nanjing Medical Center for Clinical Pharmacy, Nanjing Drum Tower Hospital, Affiliated Hospital of Medical School, Nanjing University, Nanjing, China; 2 Department of Hematology, Nanjing Drum Tower Hospital Clinical College of Nanjing University of Chinese Medicine, Nanjing, China; 3 Division of Spine Surgery, Department of Orthopedic Surgery, Nanjing Drum Tower Hospital, Affiliated Hospital of Medical School, Nanjing University, Nanjing, China; 4 Department of Endocrinology, Jinling Hospital, Medical School of Nanjing University, Nanjing, China; 5 School of Pharmacy, Nanjing University of Chinese Medicine, Nanjing, China; 6 Department of Colorectal Surgery, Nanjing Drum Tower Hospital, Affiliated Hospital of Medical School, Nanjing University, Nanjing, China

**Keywords:** Cell biology, Structural biology

## Abstract

Migrasomes are a newly discovered type of organelle, typically located at the tips or intersections of retraction fibers, containing vesicles of various sizes and numbers. During cell migration, migrasomes expand in size, are accompanied by the rupture of retraction fibers, and eventually enter the intercellular space or are absorbed by neighboring cells. Increasing research has shown that migrasomes play crucial roles in cellular growth and development, including maintaining intracellular homeostasis and facilitating intercellular communication. This review focuses on the biogenesis, functions, and pathological roles of migrasomes, while also exploring their future research prospects. As a novel mechanism of intercellular communication, migrasomes hold immense potential for therapeutic applications. A deeper understanding of how to leverage their physiological functions for disease diagnosis and treatment will be a critical focus of future investigations.

## Facts

- Migrasomes are a newly discovered type of organelle, typically located at the tips or intersections of retraction fibers, containing vesicles of various sizes and numbers.
- Maintenance of intracellular homeostasis: Regulating mitochondrial quality control, eliminating autophagosomes/endoplasmic reticulum proteins to alleviate stress, and managing metabolic imbalances.
- Facilitation of intercellular communication: Mediating the transfer of mRNA, proteins, and signaling molecules (e.g., TGFB2, IL1B, PDGFD, CXCL12) between cells, influencing processes such as embryonic development, angiogenesis, and immune responses.
- Pathological implications: Emerging roles in diseases like proliferative vitreoretinopathy (PVR), podocyte injury, viral infections, and tumor progression. For example, migrasomes promote RPE cell activation in PVR via the TGFB2 pathway and serve as carriers for viral dissemination.

## Open questions

- How can we develop efficient, non-invasive techniques for real-time imaging and purification of migrasomes to facilitate in-depth mechanistic studies?
- Do additional signaling pathways (e.g., Rho/ROCK, integrin-ECM interactions) modulate migrasome formation beyond known regulators?
- How do migrasome functions differ across physiological versus pathological contexts (e.g., immune responses, tumor microenvironments)? Are their roles in cell-cell communication context-dependent?
- Beyond releasing contents or being internalized by recipient cells, do migrasomes engage alternative mechanisms (e.g., membrane fusion, long-range signaling) to exert biological effects?

## Introduction

**Fig. 1:**
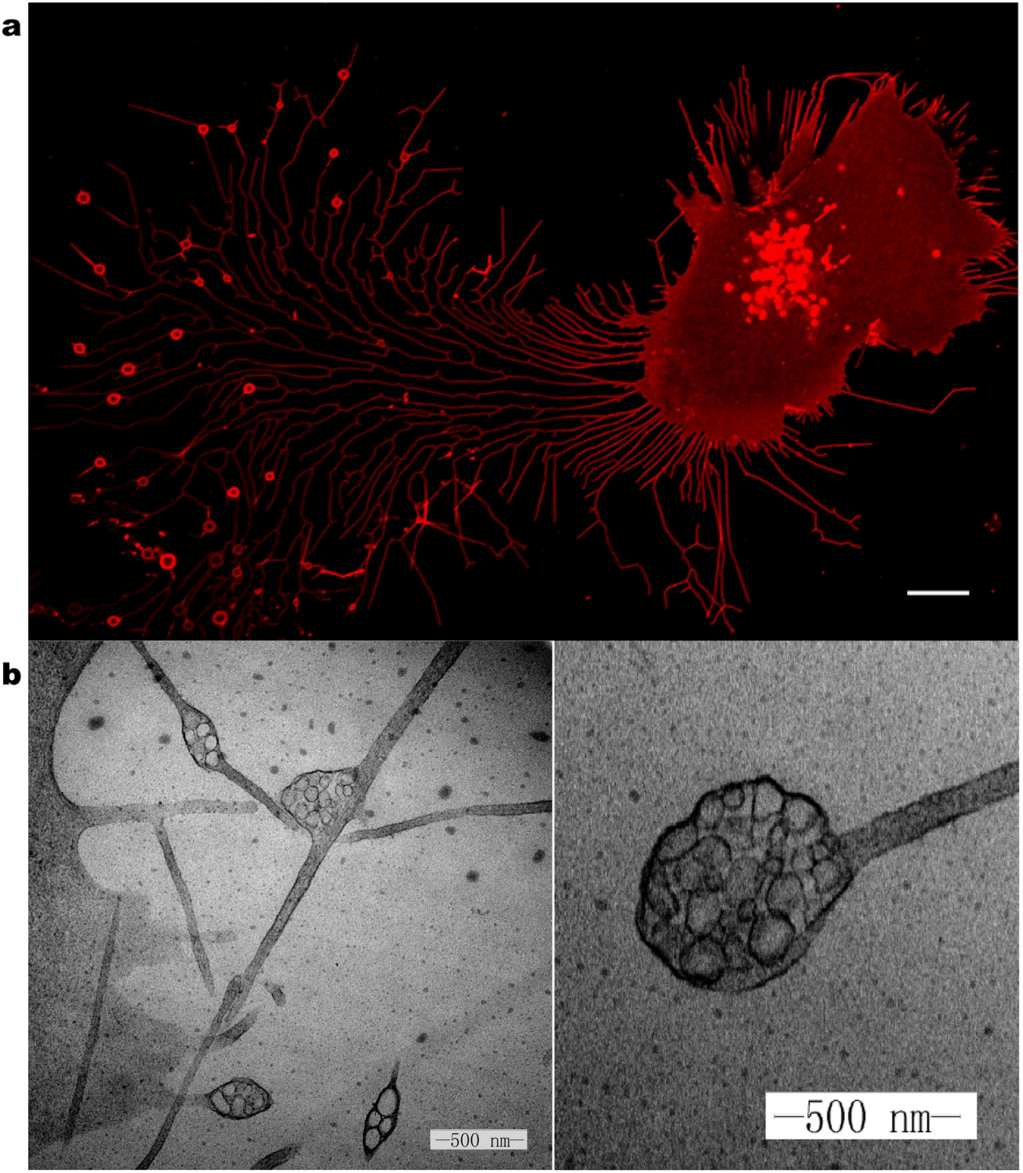
Morphology of migrasomes and their internal granular ultrastructure in L929 cells. **a** Morphology of migrasomes in L929 cells transfected with Tspan4-mCherry, visualized by confocal microscopy. Scale bar, 10 mm. **b** Transmission electron microscopy image of migrasomes, showing their internal granular ultrastructure. Scale bar, 500 nm.

Cell migration is a core mechanism in various physiological and pathological processes, including development, immune response, and tumor metastasis. In recent years, the discovery of migrasomes as a novel type of organelle has provided a new perspective for understanding the dynamic regulation and biological functions of cell migration. Migrasomes are located at the tips or intersections of contractile fibers and are rich in vesicular structures (Fig. 1) [1]. Their formation is closely related to cell migration: as cells migrate, migrasomes gradually enlarge and are released into the extracellular space or neighboring cells through the rupture of contractile fibers [1]. Studies have shown that migrasomes are not only involved in the maintenance of cellular homeostasis (such as mitochondrial quality control and clearance of stress proteins) but also play key roles in processes like embryonic development, angiogenesis, and immune regulation [2]. In addition, the association of migrasomes with various diseases (such as proliferative vitreoretinopathy, viral infections, and tumor progression) has gradually been revealed, suggesting their potential clinical value as a novel intercellular communication carrier [3]. With the development of single-cell imaging techniques and omics analysis, research on the molecular mechanisms and functions of migrasomes has become a hot topic at the intersection of cell biology and translational medicine.

Currently, significant progress has been made in the biogenesis mechanisms of migrasomes. Studies have confirmed that their formation involves multi-stage regulation: nucleation mediated by PIP5K1A/RAB35, and maturation and expansion driven by the enrichment of sphingomyelin (SM) and TSPAN4 in microdomains [4], which are also indirectly regulated by signaling pathways such as ROCK1 and integrin-extracellular matrix interactions [5–7]. At the functional level, migrasomes participate in intercellular communication and disease progression by delivering mRNA, proteins (such as TGFB2 and CXCL12), and viral particles [8]. However, there are still significant gaps in the current research: first, the functional heterogeneity of migrasomes in different physiological or pathological environments (such as the tumor microenvironment and immune response) is not yet clear; second, whether their regulatory networks depend on other signaling pathways still needs systematic validation; third, existing imaging and purification technologies cannot achieve real-time dynamic tracking and precise functional analysis of migrasomes [9], which limits their translational applications.

This article aims to systematically review the biogenesis mechanisms, physiological functions, and pathological significance of migrasomes, integrate the latest research progress, and reveal their potential as disease diagnostic biomarkers or therapeutic targets. Compared with previous reviews, the innovations of this article are mainly reflected in the following three aspects: in terms of mechanism depth, it discusses the role of tetraspanins in the contexts of migrasomes and exosomes; in terms of pathological associations, it systematically summarizes the dual roles of migrasomes in viral spread, tumor microenvironment remodeling, and organ damage, and proposes therapeutic strategies targeting migrasomes; in terms of technical challenges, it reviews the bottlenecks in imaging and separation technologies for migrasome research and looks forward to solutions driven by artificial intelligence and nanotechnology. Through multidimensional analysis, this article provides a theoretical framework for migrasome research and lays the foundation for developing precision medicine strategies based on migrasomes.

## Biogenesis of migrasomes

**Fig. 2:**
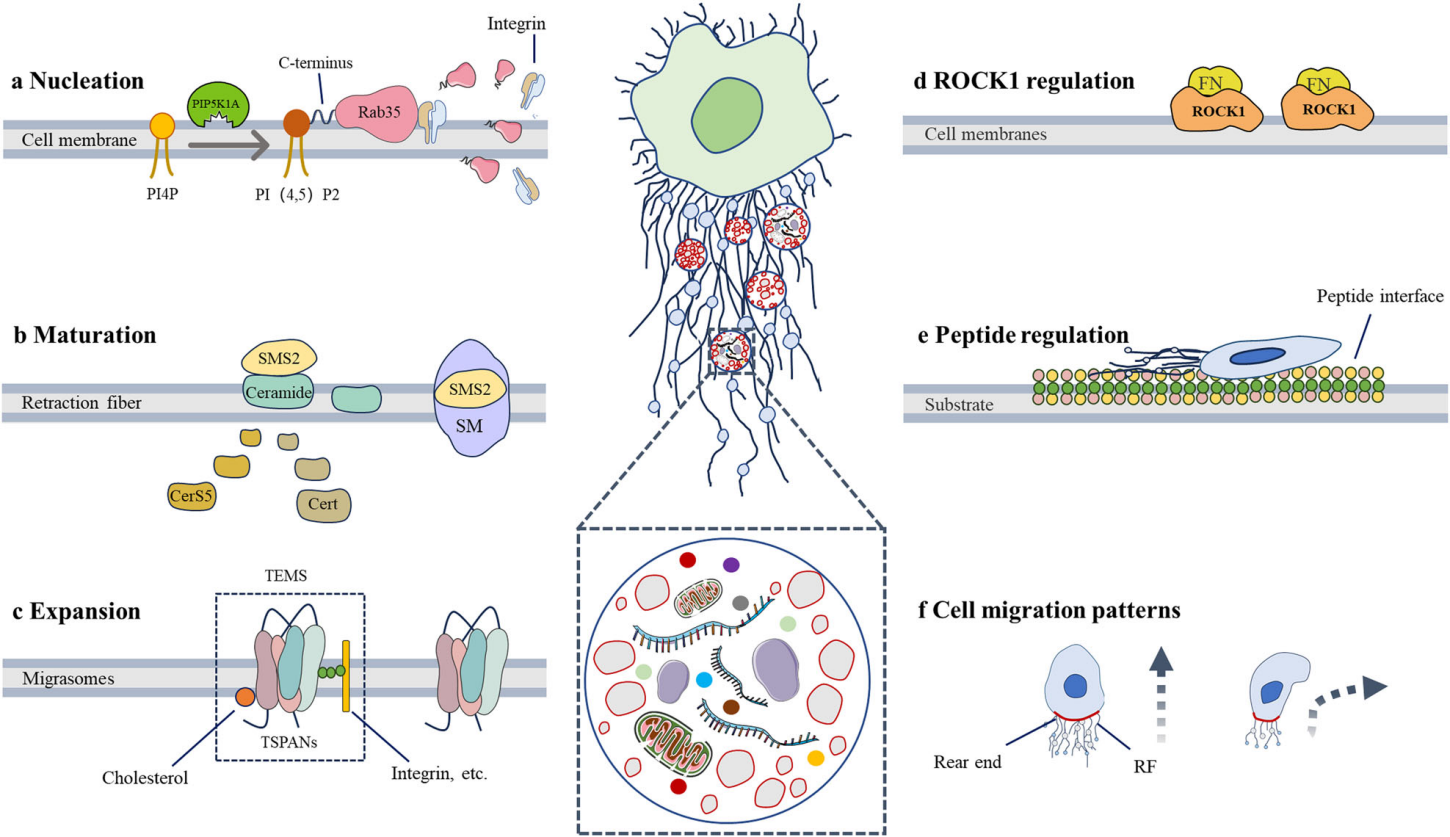
The formation of migrasomes. **a** Nucleation, PIP5K1A converts PI4P to PI(4,5)P2, PI(4,5)P2 interacts with the C-terminus of RAB35, thereby recruiting more RAB35 and integrin α5. **b** Maturation, cells gradually migrate away, while the anchored SMS2 spots remain on the retraction fibers, becoming the formation site of migrasomes. At the SMS2 spots, ceramide is converted into sphingolipids, thus promoting the growth of migrasomes. **c** Amplification, TSPAN and other proteins, integrins, cholesterol combine to form TSPAN4-enriched microdomains. **d** ROCK1 interacts with fibronectin, regulating the formation of migrasomes. **e** Peptide-modified surfaces promote the formation of longer retraction fibers and more migrasomes by cells. **f** Cell migration affects the formation of migrasomes by regulating the number and length of RFs.

The biogenesis of migrasomes is closely linked to cell migration, meaning that any factor affecting cell migration may directly or indirectly regulate the formation of migrasomes (Fig. 2). Several factors, including *PIP5K1A*, RAB35, ITGA5, ITGB1, sphingomyelin (SM), sphingomyelin synthase 2 (SMS2), and TSPAN4, are directly involved in the nucleation, maturation, and expansion of migrasomes. In addition, Rho-associated kinase 1 (ROCK1), peptide-modified substrates, and the density of RFs exerts indirect regulatory effects on migrasome biogenesis through various signaling mechanisms.

### Migrasomes formation

The formation of migrasomes is a complex and highly regulated process, orchestrated by multiple signaling pathways and typically divided into three distinct stages: nucleation, maturation, and amplification [4]. Each stage involves the action of distinct proteins and signaling pathways. During the nucleation stage, factors such as PIP5K1A, RAB35, ITGA5, and ITGB1 are involved in regulation. In the maturation stage, SM and SMS2 play crucial roles. Finally, the formation of TSPAN4-enriched microdomains (TEMs) by TSPAN4 self-assembly or integration with other transmembrane proteins or cytoplasmic proteins during the amplification phase is vital for further expansion and stabilization of migrasomes.

#### Nucleation

PIP5K1A, a phosphatidylinositol 4-phosphate (PI4P) kinase, plays a critical role in the nucleation of migrasomes by converting PI4P to PI(4,5)P2, which is then recruited to the site of migrasomes formation. The accumulation of PI(4,5)P2 at this site further facilitates its interaction with the C-terminal polybasic cluster of RAB35, thereby promoting the recruitment of additional RAB35 molecules. Activated RAB35, in turn, recruits integrin α5 to the migrasome formation site, acting as an initial signal for migrasome biogenesis [10, 11].

Integrins are heterodimeric transmembrane receptors composed of α and β subunits, which are expressed across various cell types and mediate binding to specific extracellular matrix (ECM) proteins or other extracellular receptors. Eighteen α subunits and eight β subunits are known in mammals, and these subunits can combine in various configurations to form 24 distinct integrins [12]. Wu et al. demonstrated that integrins in different cell types bind to different ECM proteins, influencing the formation of migrasomes [13]. For example, in rat renal cells (NRK), integrin α5β1 accumulates at the base of migrasomes, and knockdown of the ITGA5 gene, which encodes the α5 subunit, inhibits migrasomes formation in cells cultured on fibronectin. In CHO-GFP cells, overexpression of integrin α1 enhances migrasomes formation on collagen IV, suggesting a high-affinity interaction, though integrin α1 does not exert the same effect on other ECM proteins. Similarly, in MGC803 cells, overexpression of integrins α3 and α1, along with their respective ECM partner proteins, also promotes migrasome formation. These findings underscore the critical role of integrin-ECM interactions in the regulation of migrasomes biogenesis.

#### Maturation

SM is one of the most abundant lipids in the plasma membrane. SM is synthesized by SMS, which transfers phosphocholine from phosphatidylcholine to the C-1 hydroxyl group of ceramide. In mammalian cells, two isoforms of sphingomyelin synthase are present: sphingomyelin synthase 1 (SMS1) and SMS2. Liang et al. demonstrated that both sphingomyelin and ceramide are critical for migrasome formation [14]. SM is enriched within migrasomes, and hydrolysis of SM significantly reduces the number of migrasomes, which disappear shortly after SM hydrolysis. SMS2 is concentrated at the basal membrane at the leading edge of the cell, where it assembles into fixed sites. As the cell migrates, SMS2 sites gradually “move out” of the cell, entering RFs and becoming the nucleation sites for migrasomes formation, eventually maturing into fully formed migrasomes. Additionally, ceramide synthase 5 (CERS5), required for ceramide synthesis, and ceramide transfer protein (CERT), which transports ceramide, are both essential for migrasome formation. Knockdown of either CERS5 or CERT results in the blockade of migrasomes formation [14].

#### Amplification

TSPAN4 is a highly conserved protein family that contains four transmembrane domains (TM1, TM2, TM3, and TM4), two extracellular loops (SEL and LEL), and both N- and C-terminal regions. The SEL loop connects TM1 and TM2, while the LEL loop connects TM3 and TM4 [15]. In mammals, the tetraspanin family consists of 33 members, numbered TSPAN1 to TSPAN33. Some of these members have commonly recognized names; for example, TSPAN29, TSPAN24, and TSPAN28 correspond to CD9, CD151, and CD81, respectively [16].

Huang et al. screened 33 tetraspanins (TSPANs) for their overexpression effects on migrasome formation [17]. The results indicated that overexpression of 14 of these TSPAN proteins could promote migrasome formation. Using CRISPR-Cas9 technology to knockout TSPAN4, the authors found that the depletion of TSPAN4 significantly inhibited migrasome formation in both MGC-803 and NRK cells, further confirming the crucial role of TSPAN4 in migrasome generation [17]. However, it is important to note that knocking out TSPAN4 in L929 cells also affected migrasome formation, leading the authors to speculate that other tetraspanins in L929 cells might compensate for the loss of TSPAN4 in regulating migrasome formation [17]. This suggests that the TSPAN proteins involved in migrasome regulation may vary across different cell types. Additionally, Zimmerman et al. discovered that CD81 contains a cholesterol-binding pocket, which aids in the specific interaction between TSPAN4 and cholesterol [18]. Migrasomes are rich in cholesterol, and its critical role in migrasome formation has been well-established. Cholesterol, in combination with TSPAN4, forms the tetraspanin-enriched microdomain, and multiple TSPAN4-enriched microdomains aggregate to form larger structural units [17, 19]. In experimental settings, treatment with the cholesterol-depleting agent methyl-β-cyclodextrin significantly inhibited migrasome formation. Furthermore, the authors demonstrated in an in vitro membrane system that TSPAN4 and cholesterol alone are sufficient to reconstruct migrasomes [17].

It should be specifically pointed out that CD9 (TSPAN29) and CD81 (TSPAN28) are also signature proteins of exosomes [20], but they play different functions in the two types of vesicles. In migrasomes, CD9 and CD81 promote the expansion of migrasomes by forming TEMAs and stabilize the structure of migrasomes by increasing membrane bending rigidity [21]. In exosomes, CD9 and CD81 often participate in the sorting and release of exosomes through interactions with the endosomal sorting complexes required for transport (ESCRT) mechanism during the formation and secretion of exosomes [20]. In addition, CD9 and CD81 also affect the gene expression and signaling pathways of target cells through exosome-mediated intercellular communication [20]. These differences indicate that CD9 and CD81 play different functions in migrasomes and exosomes.

### Other factors influencing migrasome formation

In addition to the aforementioned proteins and signaling pathways, such as PI4P kinase, RAB35, SM, integrins, and TSPAN4, several other factors also influence migrasome formation. For instance, ROCK1, peptide-modifying proteins, and the direction and speed of cell migration have been shown to affect migrasome generation. ROCK1 primarily regulates the interaction between cells and fibronectin, thereby influencing migrasome formation [5].

#### ROCK1 regulation of migrasome formation

Rho GTPases, members of the Ras superfamily, share approximately 25% homology with Ras. The major members of Rho GTPases include RhoA, RhoB, and RhoC [22]. Rho-associated kinase (ROCK), a key downstream effector of Rho, is one of the most extensively studied molecules in functional research. The Rho/ROCK signaling pathway induces cytoskeletal rearrangement, cell migration, and stress fiber formation [22]. ROCK plays a crucial role in the formation of migrasomes by regulating the adhesion between cells and fibronectin. Lu et al. screened compounds and their protein targets that regulate migrasome formation, identifying the ROCK1 inhibitor SAR407899 as a potent suppressor of migrasome formation [5]. Further research elucidated the pivotal role of ROCK1 in migrasome generation, demonstrating that ROCK1 regulates the cell’s adhesion to fibronectin and traction forces during this process [5]. Specifically, cells with low ROCK1 expression displayed reduced adhesion and significantly diminished traction force when exposed to increased concentrations of fibronectin, impairing normal migrasome formation compared to wild-type cells [5]. These findings underscore the central role of ROCK1 in cell adhesion and traction force generation, which in turn regulates migrasome formation.

#### Effects of peptide-modified substrates on migrasome formation

Peptides, composed of various amino acids, are fundamental building blocks of proteins. Due to their excellent biocompatibility, low immunogenicity, biodegradability, and biological activity, peptides have found widespread applications in the biomedical field [23]. Research by Saito et al. demonstrated that peptide-modified interfaces, composed of cell-penetrating peptides (pVEC and R9) and viral fusion peptides (SIV), outperformed the use of fibronectin, integrin-binding peptides (RGD), or unmodified substrates in promoting cell migration and migrasome formation [6]. These specific peptide combinations significantly enhance the efficiency of cell migration and migrasome formation by providing a biologically active interface.

#### Coordinated role of cell migration

Cell migration is a fundamental process that occurs widely in numerous physiological and pathological events, including growth and development, wound healing, and tumor metastasis. It is a complex process intricately regulated by various signaling pathways. The formation of migrasomes is typically dependent on cell migration. In exploring how cell migration influences migrasome formation, Fan et al. found that cell migration affected migrasome formation by regulating the number and length of RFs [7]. Specifically, when cells change direction during migration, the number of RFs decreases due to cell rear contraction, leading to fewer migrasomes. In contrast, during linear migration with faster cell movement, more and longer RFs are formed, which in turn promotes the formation of migrasomes.

## Biological functions of migrasomes

Migrasomes play multifaceted key roles in maintaining cellular homeostasis and facilitating intercellular communication. Migrasomes mediate a specialized mechanism of mitochondrial quality control, where damaged mitochondria are transported to the cell periphery by motor proteins (such as KIF5B and Myo19) and are eliminated via migrasomes. This process is distinct from the canonical mitophagy pathways found in post-mitotic cells like neurons. In addition to mitochondrial regulation, migrasomes act as a key platform for cellular homeostasis by expelling stress-related components, such as autophagosomes and endoplasmic reticulum proteins, thereby alleviating metabolic imbalances and endoplasmic reticulum stress. Moreover, migrasomes serve as mediators of intercellular communication by transferring bioactive molecules (including mRNA and proteins) and signaling molecules to recipient cells, influencing physiological processes such as embryonic development and organ formation, angiogenesis, and more. Their role in embryogenesis was exemplified in zebrafish studies, where migrasome-enriched signaling molecules guided the migration of dorsal forerunner cells and the formation of left-right asymmetry. In mammals, monocyte-derived migrasomes drive vascular network expansion through chemokine-mediated recruitment. Although migrasomes share similarities with exosomes in terms of molecular transfer, they have unique mechanisms of formation and functional adaptations related to cell motility. Collectively, these findings highlight the role of migrasomes as dynamic regulators, offering new insights into therapeutic strategies for diseases associated with cellular homeostasis or intercellular communication dysregulation.

### The role of migrasomes in the maintenance of cellular homeostasis

#### Mitochondrial quality control

**Fig. 3:**
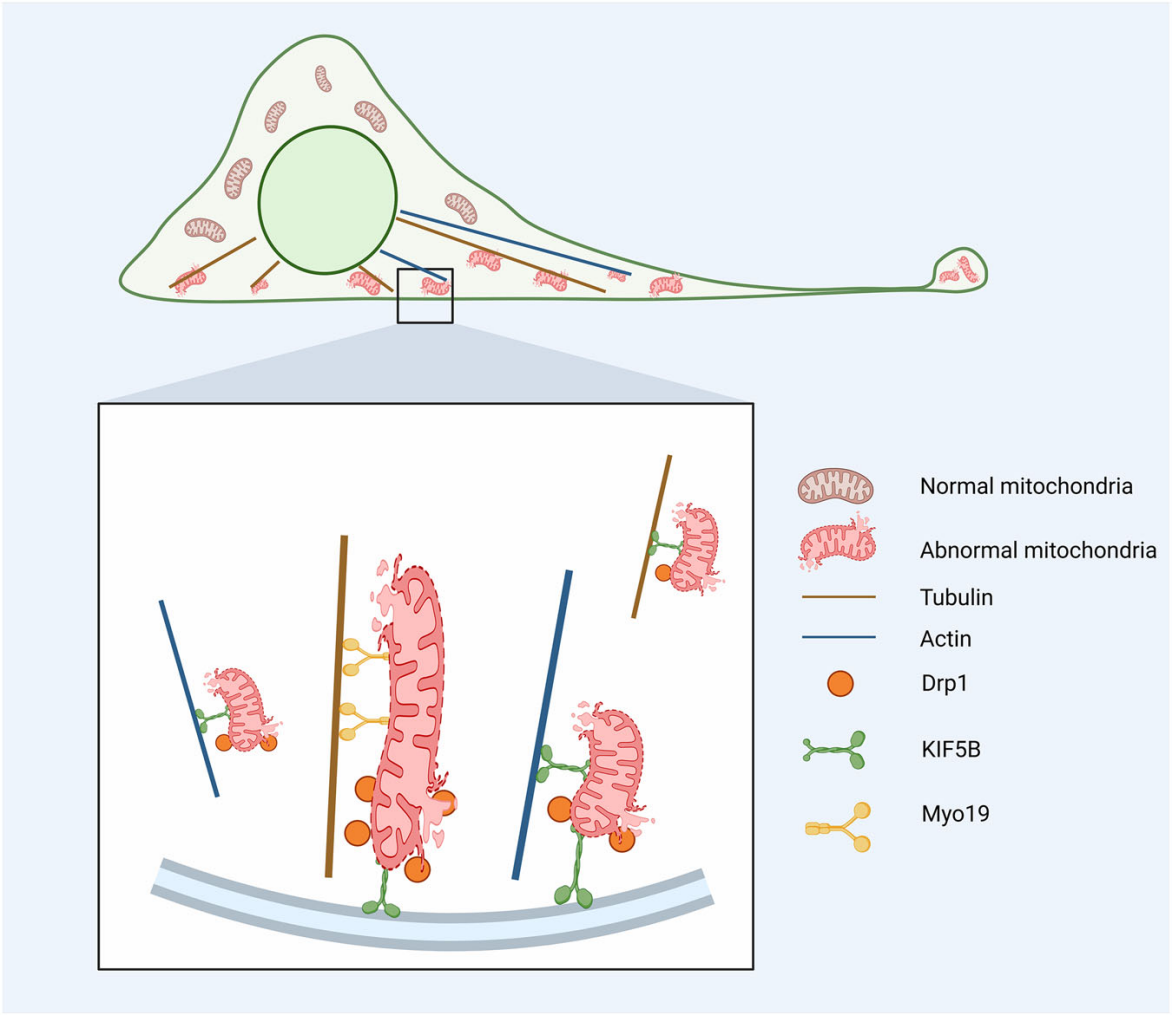
KIF5B, Drp1, and Myosin19 are involved in the removal of damaged mitochondria from the cell. Created in https://BioRender.com.

Mitochondria are key organelles responsible for essential cellular functions such as energy metabolism, proliferation, differentiation, immune response, and redox balance. To maintain their normal function and structure, cells have established a complex mitochondrial quality control system, which includes processes such as mitochondrial biogenesis, dynamic regulation (fusion and fission), and mitophagy [8]. Furthermore, migrasome-mediated mitochondrial exocytosis has been recognized as an emerging mechanism of mitochondrial quality control. The research by Jiao et al. confirmed this process in macrophages and murine neutrophils [24]. When mitochondria are damaged, they bind with motor proteins on the exterior of cells and are subsequently localized to the cell periphery. During migrasome formation, mitochondria extend outward through tubular structures, undergo division, and produce fragments from the mitochondrial network. These fragments adhere to the cell membrane, remain on the RFs as the cell migrates, and eventually become incorporated into migrasomes. As migrasomes accumulate, the damaged mitochondria are effectively cleared from the cell [24]. This process depends on the coordinated actions of motor protein family members, including KIF5B, the dynamin-related protein Drp1, and myosin Myo19. These proteins finely regulate mitochondrial transport, localization, and division, collectively facilitating mitochondrial exocytosis and efficiently removing damaged mitochondria to maintain mitochondrial homeostasis within the cell (Fig. 3) [24].

Notably, while migrasome-mediated exocytosis represents a significant pathway for damaged mitochondria clearance in motile cells, recent studies suggest that not all cells with mitochondrial damage necessarily undergo migration or utilize migrasomes. For instance, post-mitotic cells such as neurons and cardiomyocytes—which exhibit limited migratory capacity—appear to rely more heavily on canonical mitophagy pathways for mitochondrial quality control [25–28]. Additionally, certain stressed cells may prioritize other quality control mechanisms, such as sequestering dysfunctional mitochondria into perinuclear aggregates or triggering apoptosis when damage exceeds a critical threshold [28]. This heterogeneity in response underscores the existence of context-dependent strategies in mitochondrial quality regulation, where migrasome utilization represents one specialized mechanism among multiple cellular adaptation options.

#### Exclusion of autophagosomes and endoplasmic reticulum proteins

The relationship between migrasomes and cellular homeostasis is mainly reflected in their ability to help cells cope with various stress conditions and maintain internal stability by excluding specific substances. Autophagosomes are key structures in the autophagy process, responsible for wrapping and transporting damaged components within the cell (such as protein aggregates and damaged organelles) to lysosomes for degradation [29]. Studies by Lee et al. have shown that migrasomes help cells exclude damaged substances and stress-related molecules by carrying autophagosomes and endoplasmic reticulum proteins, thus playing an important role in maintaining cellular homeostasis [30]. The exclusion of autophagosomes helps cells maintain vitality under conditions of nutrient deprivation and protein homeostasis imbalance, while the exclusion of endoplasmic reticulum proteins helps alleviate endoplasmic reticulum stress [30]. Inhibition of migrasome formation weakens the cell’s ability to cope with stress, making cells more prone to death [30]. Therefore, migrasomes are not only a physical product of cell migration but may also serve as a dynamic mechanism for regulating the internal cellular environment, providing support for cell survival in complex environments.

### The role of migrasomes in cell communication

In recent years, studies have confirmed that migrasomes carry a variety of biomolecules, including mRNA, proteins, TGFB2, IL1B, PDGFD, and CXCL12. Research by Zhu et al. has shown that migrasomes contain mRNA and proteins, which can be transferred to recipient cells and translated into functional proteins, thereby affecting the function of recipient cells. For example, Pten mRNA in migrasomes can be transferred to recipient cells, translated into Pten protein, and subsequently inhibit the proliferation of cancer cells [8]. Exosomes, which are recognized as intercellular communication mediators [31], also have the ability to transfer mRNA and proteins [32]. The role of exosomes in horizontal transfer has been extensively studied; they can effectively deliver mRNA and proteins to recipient cells, thereby altering the function of recipient cells and participating in various biological processes such as immune response regulation [33], tumor invasion [34], and cell differentiation [35]. Migrasomes and exosomes share certain similarities in intercellular communication, such as their ability to carry mRNA and proteins and facilitate intercellular communication. However, there are significant differences between the two in terms of formation mechanisms, composition, and function. The formation of migrasomes is closely related to cell migration, while the formation of exosomes involves complex mechanisms of multivesicular bodies. Exosomes have a more complex composition [36] and more diverse functions [37]. Given the similarities and differences between migrasomes and exosomes in horizontal transfer, further research into their specific roles and interrelationships in intercellular communication will help to better understand these processes and provide new research directions for biomedical applications.

Jiang et al. found that migrasomes in zebrafish gastrula-stage cells are enriched with various signaling molecules [38]. Among these, the chemokines CXCL12A and CXCL12B are enriched in embryonic migrasomes and play important roles in organ morphogenesis [38]. In addition, in TSPAN4-knockout zebrafish embryos, the formation of migrasomes is significantly reduced, causing the dorsal forerunner cells (DFCs) to disperse during migration and fail to reach their destination [38]. This, in turn, affects the formation of Kupffer’s vesicle and the development of the embryo’s left-right asymmetry [38]. This further confirms the key role of migrasomes in ensuring the normal progression of embryonic development and the proper formation of organs.

**Fig. 4:**
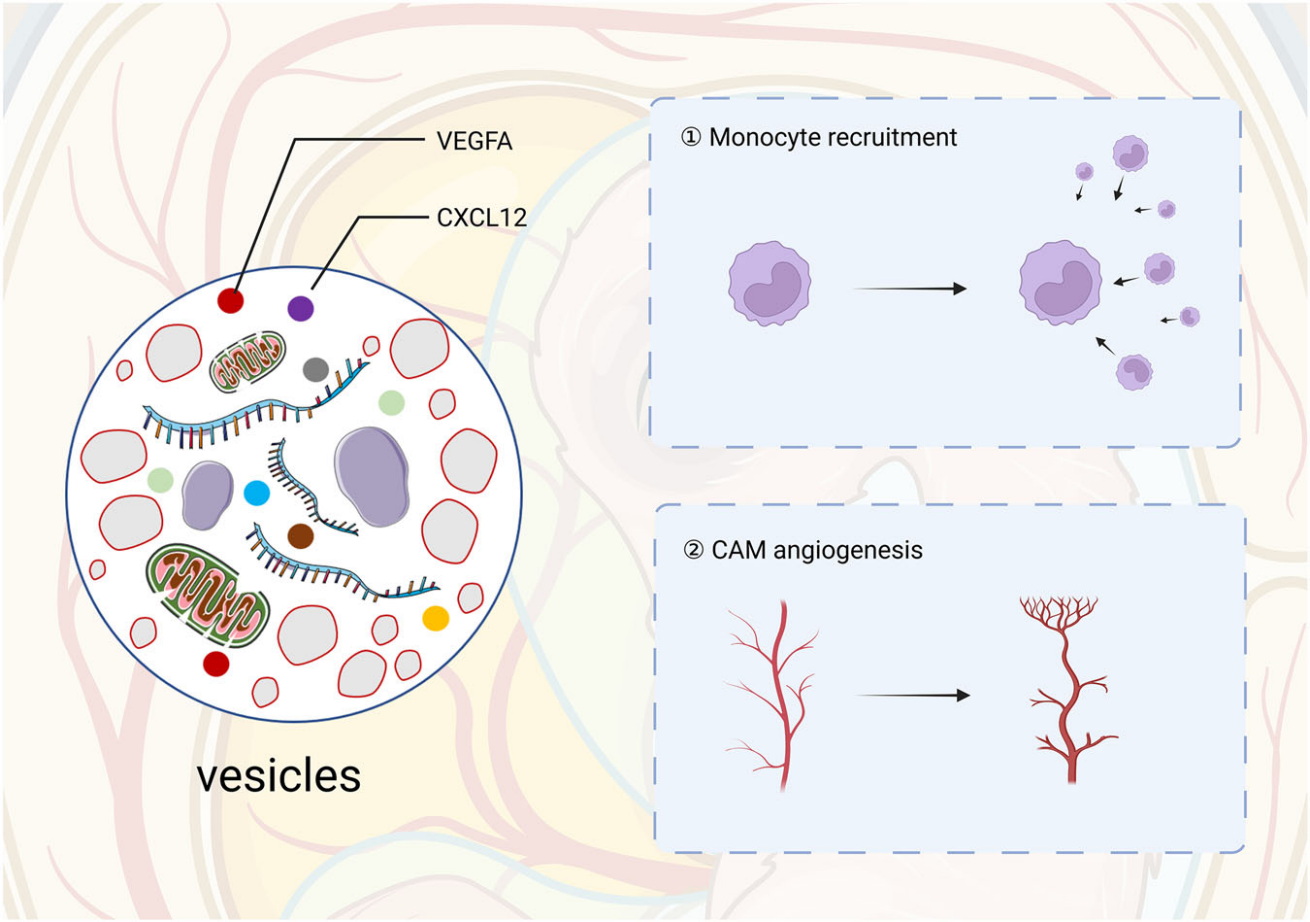
Illustration of migrasomes from monocytes in angiogenesis during embryonic development. Created in https://BioRender.com.

Zhang et al. demonstrated that monocytes deposit migrasomes enriched with VEGFA and CXCL12 to drive embryonic angiogenesis (Fig. 4) [35]. These migrasomes recruit additional monocytes via CXCL12-mediated chemotaxis, forming a self-amplifying loop that accelerates capillary network formation [35].

## Pathological role of migrasomes

Given their involvement in various crucial physiological functions, migrasomes also participate in pathological processes under certain conditions. As mentioned earlier, migrasomes regulate vascular homeostasis, but during the pathological process of sodium chloride-induced acute ischemic stroke, migrasomes exacerbate ischemic cell injury by interacting with the cytoplasm of surrounding neuronal cells [39]. Additionally, migrasomes are closely associated with several diseases, including proliferative vitreoretinopathy (PVR), podocyte injury, viral spread, and cancer.

### Migrasomes and proliferative vitreoretinopathy

Proliferative vitreoretinopathy (PVR) is a pathological response to retinal damage (such as retinal detachment or abnormal healing after retinal detachment surgery), characterized by the proliferation of retinal pigment epithelial (RPE) cells [40]. The mechanism underlying PVR is complex and resembles an abnormal wound healing process. In addition to RPE cells, inflammatory cells, retinal cells, and various cytokines play critical roles in its development. Surgery is currently the primary treatment; however, the therapeutic outcomes are often suboptimal. Studies have shown that multiple signaling pathways are involved in regulating the formation of PVR, including the NF-κB pathway, MAPK and its downstream pathways, JAK/STAT signaling, PI3K/Akt signaling, thrombin and its receptor pathways, TGFB and its downstream signaling, Notch signaling, and Wnt/β-catenin signaling [34]. Among these, TGFB, a multifunctional cytokine, regulates several key biological processes such as differentiation, apoptosis, and migration. Previous research has indicated that TGFB is overexpressed in the vitreous of PVR patients and is closely associated with the severity of PVR [41].

**Fig. 5:**
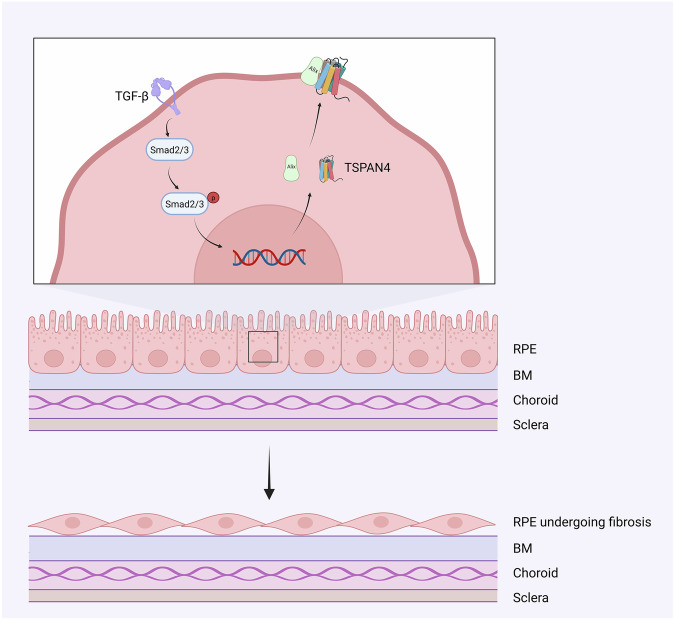
Illustration of migrasomes characteristics in proliferative vitreoretinopathy (PVR). Created in https://BioRender.com.

Wu et al. revealed the pivotal role of migrasomes in the activation of retinal pigment epithelial (RPE) cells during PVR (Fig. 5) [42]. Their study demonstrated that during RPE cell activation, TGFB1 expression increases, driving TSPAN4 upregulation and subsequent migrasome formation. The presence of migrasomes in retinal Müller cells was first confirmed through the migrasomes marker TSPAN4 [42]. Further regulation of TSPAN4 expression both in vitro and in vivo confirmed that migrasomes contributed to RPE cell activation and the progression of PVR. In the PVR microenvironment, exposure of RPE cells to TGFB activated the Smad2/3 signaling pathway, which subsequently triggered TSPAN4 expression and migrasome formation. Blocking migrasome formation in the PVR microenvironment may represent one potential therapeutic approach for PVR, although whether this intervention would affect other normal cellular physiological functions warrants further investigation.

### Migrasomes and podocyte injury

Podocytes are essential cells responsible for maintaining the glomerular filtration barrier function. They are capable of adapting to and sustaining functional balance in response to appropriate physiological pressure or pathological stimuli. Podocytes possess intrinsic regulatory mechanisms to withstand stress; however, when external pressures exceed their regulatory capacity, “podocyte injury” occurs. Electron microscopy reveals morphological changes in podocytes, including the disappearance of foot processes, formation of cytoplasmic vacuoles, bubble phenomena, and irregularities in organelle and cell membrane morphology [43]. Proteinuria is an early consequence of podocyte injury and a hallmark sign of kidney disease, often included as a diagnostic criterion for renal injury. Since podocytes are terminally differentiated cells with minimal proliferative capacity [44], early detection of podocyte injury is crucial for preventing the progression of glomerular diseases [45].

Liu et al. demonstrated that podocyte injury induced by lipopolysaccharide (LPS), puromycin aminonucleoside (PAN), or high glucose (HG) significantly increases migrasome secretion from human or murine podocytes [46]. Notably, during PAN nephropathy, the increase in urinary migrasome levels increase earlier than the elevation of proteinuria, suggesting that urinary migrasomes may serve as a more sensitive early marker of podocyte injury compared to proteinuria.

### Migrasomes and promotion of viral spread

**Fig. 6:**
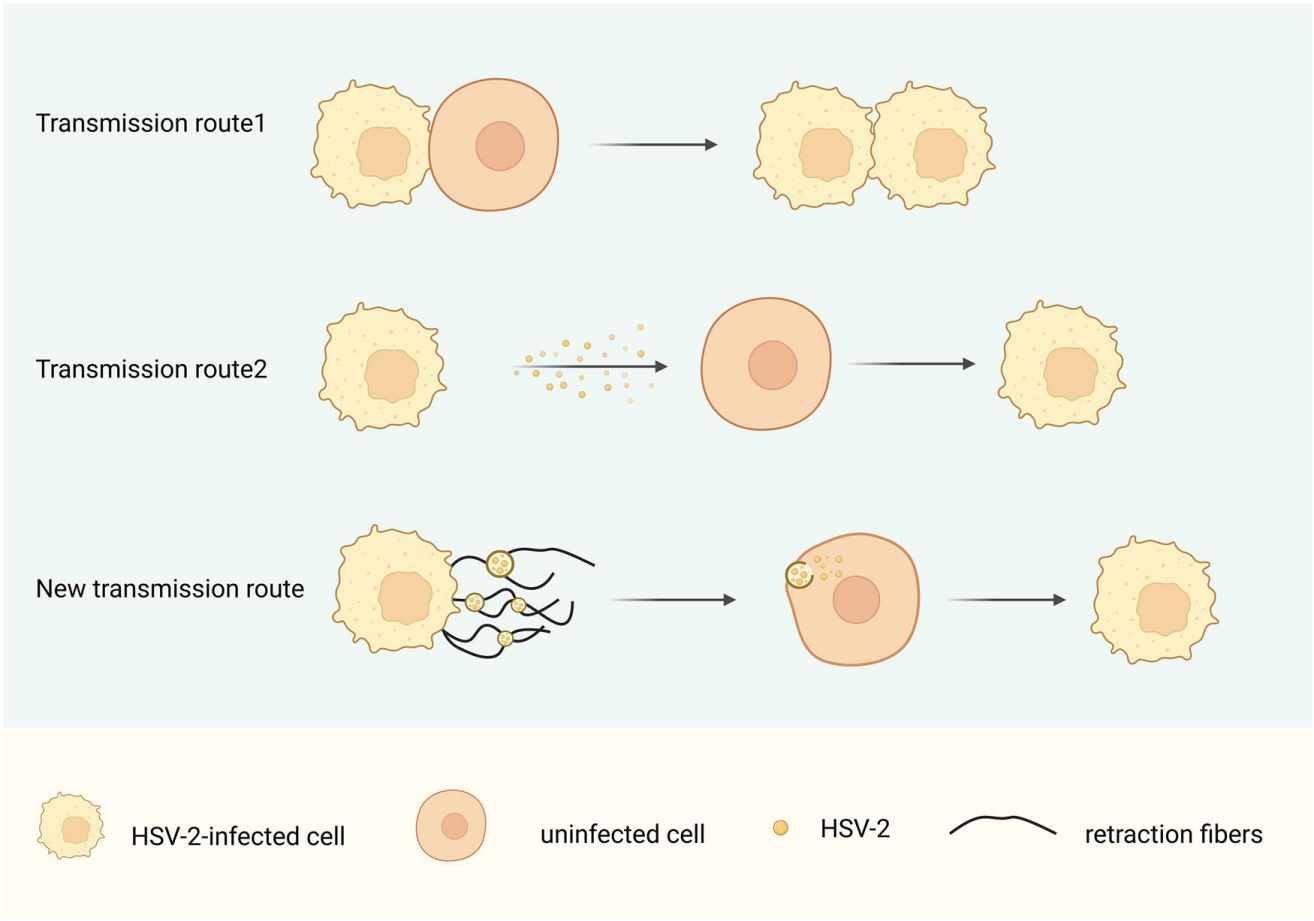
Schematic diagram of cell-to-cell transmission of HSV-2 using migrasomes. Created in https://BioRender.com.

Zhang et al. observed the presence of migrasomes in cells infected with Chikungunya virus (CHIKV) and discovered a strong co-localization of the viral protein NSP1 with ITGB1 in infected cells [47]. By knocking down ITGB1, the researchers found a significant reduction in CHIKV RNA levels. Furthermore, they identified an interaction between NSP1 and *PIP5K1A*, further confirming that CHIKV induces migrasome formation [47]. Lv et al. demonstrated that cells infected with Vaccinia virus (VACV), the prototype virus of the poxvirus family, induce migrasome formation in the late stages of infection [48]. Since poxvirus spread typically relies on extracellular enveloped virus (EEV), and migrasomes contain intracellular mature virus (IMV) and intracellular enveloped virus (IEV), this suggests that migrasomes may represent a novel mechanism for poxvirus transmission [48]. Liu et al. found that migrasomes serve as carriers for viral spread in cells infected with Herpes simplex virus type 2 (HSV-2) [49]. Upon purifying the contents of migrasomes from HSV-2-infected HaCaT cells, the researchers found HSV-2 viral particles, which were capable of spreading the infection (Fig. 6) [49]. Therefore, blocking migrasome formation in infected cells could represent a potential new strategy for antiviral therapy (Table 1).

**Table 1:** Functions of migrasomes in virus transmission.

Virus	Functions	Reference
CHIKV	co-localization of NSP1 and ITGB1; interaction of nsP1 with *PIP5K1A*	[47]
VACV	migrasomes contain IMV, IEV	[48]
HSV-2	migrasomes contain HSV-2	[49]

### The impact of migrasomes on tumors

Cancer remains a major challenge in global public health and has become one of the key factors influencing human health. Tumors are often associated with local or distant metastasis, which disrupts the structure and function of vital organs, increasing the risk of mortality. Due to the high metabolic activity and rapid growth characteristics of tumor cells, they are capable of producing large amounts of migrasomes during proliferation or migration, which in turn influence the progression of various cancers. Additionally, migrasome-associated proteins, such as NDST1, EOGT, and PIGK, have been shown to affect tumor growth and patient survival (Table 2) [50].

**Table 2:** Functions of migrasome in different cancers.

Cancer	Functions	Reference
pancreatic cancer	promote tumor cell proliferation and invasion	[51]
hepatocellular Carcinoma (HCC), lung cancer, colorectal cancer, prostate cancer	CD151 as unfavorable prognostic indicator	[52–55]
breast cancer, gastric carcinoma, renal carcinoma	NDST1/EOGT/PIGK as unfavorable prognostic indicators or promotion of tumor cell proliferation and migration	[57–59]

Pancreatic cancer is a highly malignant gastrointestinal tumor, characterized by insidious onset, rapid progression, and severe malignancy. The tumor microenvironment is often characterized by substantial infiltration of inflammatory immune cells and fibrosis, which are distinctive pathological features of pancreatic cancer. Zhang et al. found that during migration, pancreatic cancer cells release large amounts of migrasomes rich in immune microenvironmental regulators, such as CXCL5, TGFB1, integrin pathway proteins, and Rab family proteins [51]. These migrasomes, once absorbed by macrophages, induce M2 polarization, thereby promoting tumor cell proliferation and invasion [51].

Hepatocellular carcinoma (HCC) is one of the most lethal malignant tumors, with high metastatic potential and extensive vascularization, which makes treatment challenging and results in poor prognosis. Zhang et al. demonstrated that CD151 facilitates liver cancer cell metastasis and invasion via migrasomes, with migrasomes playing a crucial role in the vascular remodeling process of tumor cells [52]. As a member of the TSPAN4 family, the critical role of CD151 in angiogenesis and cancer metastasis has been well established and is closely associated with various highly invasive cancers, such as lung cancer [53, 54], colorectal cancer [54], and prostate cancer [55].

However, the interaction between migrasomes and the tumor suppressor Pten suggests that the influence of migrasomes on tumors is not singular. Pten protein exerts its tumor-suppressing function by dephosphorylating phosphatidylinositol-3,4,5-triphosphate (PIP3) at the 3′ position, converting it to phosphatidylinositol-4,5-bisphosphate (PIP2), thereby inhibiting tumor progression [8, 56].

## Conclusions and perspectives

Migrasomes have demonstrated remarkable potential in both diagnostic and therapeutic applications. In the diagnostic field, Liu et al. reported that migrasomes in urine are more sensitive than proteinuria and can serve as an early marker for podocyte injury, offering promise for early-stage diagnosis of kidney diseases [46]. In therapeutic contexts, blocking migrasome formation may represent a novel treatment strategy. For example, research suggests that inhibiting migrasome formation could have therapeutic implications for pathological processes such as proliferative vitreoretinopathy (PVR) [42], viral transmission [57–59], and tumor progression [51, 52]. These findings point to the potential clinical application of migrasome-targeted therapies. Bone is one of the most common metastatic sites for tumors. Gu et al. designed a tetracycline-modified nanoliposome encapsulating sodium bicarbonate and sodium phosphate to target osteoclast subtypes involved in bone metastasis caused by tumor migrasome-mediated cytoplasmic transfer [60]. This approach inhibits migrasome formation and induces tumor cell death by disrupting cell membranes, thus achieving early prevention of bone metastasis [61]. Cheng et al. proposed a new strategy for the development of anti-metastatic nanomedicines by reducing the affinity of nanoparticles for extracellular migrasomes and RFs, thereby inhibiting tumor cell migration [62].

However, one of the major challenges in current migrasome research is the lack of efficient and convenient observation techniques. Live-cell imaging of migrasomes primarily relies on molecular targeting strategies, with specific markers labeled and visualized using total internal reflection fluorescence (TIRF) microscopy or real-time dynamic imaging systems [9]. Commonly used molecular targets include TSPAN4 [63], integrins [13], NDST1, PIGK, CPQ, and EOGT [64], which enable precise tracking of migrasome dynamics during cell migration. Notably, Chen et al. identified wheat germ agglutinin (WGA) as a highly specific fluorescent probe for migrasome detection [65]. WGA selectively binds to sialic acid residues on migrasome membranes, enabling rapid and efficient labeling [65]. In addition, Jing et al. developed a polyacrylic acid-coated quantum dot-based artificial antigen (FAA) that, when combined with single-particle tracking technology, achieved super-resolution imaging of membrane fiber networks [66]. The unique photophysical properties of quantum dots, including narrow emission spectra and photostability against photobleaching, make them an ideal choice for long-term real-time imaging of migrasome dynamics [66]. These technological advancements collectively enhance the spatiotemporal resolution and duration of migrasome visualization at the in vitro cellular level, providing critical insights into their regulatory mechanisms and functional roles in cellular processes such as cell migration and intercellular communication. In recent years, breakthroughs in novel microscopic technologies have significantly advanced the in vivo dynamic observation of migrasomes. Digital adaptive optics scanning light field tomography (DAOSLIMIT) integrates light field microscopy with digital adaptive optics algorithms [67]. By employing periodic scanning and multi-view data acquisition, it achieves millisecond-level 3D subcellular resolution and supports continuous imaging for tens of hours [67]. Its phase space deconvolution algorithm effectively suppresses tissue aberrations and phototoxicity, and has been successfully applied to in vivo studies of mouse spleen, liver, and zebrafish embryos [67]. Two-photon synthetic aperture microscopy (2pSAM), based on the principle of synthetic aperture radar, utilizes needle-like beam scanning and phase correlation reconstruction technology to achieve high-resolution imaging in deep tissues (>100 μm) [68]. It also corrects aberrations through multi-angle projection, maintains low phototoxicity, and supports dynamic observation for several hours, successfully resolving the interaction between neutrophils and microglia after traumatic brain injury and the formation of germinal centers [68]. In vivo imaging technologies have revealed the key roles of migrasomes in immune regulation and disease. DAOSLIMIT first uncovered the dynamic maturation mechanism of migrasomes during neutrophil migration, identified the gradient enrichment of membrane protein Ly-6G, and confirmed that migrasomes can shed through the vascular wall into the circulatory system and be taken up by distant neutrophils, indicating their function in mediating intercellular communication [67]. 2pSAM captured in tumor models the release of vesicle-like structures from circulating tumor cells (CTCs) under the influence of blood flow shear forces, which may carry signaling molecules involved in shaping the pre-metastatic microenvironment in distant organs [68]. It also revealed the dynamic process of peripheral neutrophils migrating to the central nervous system and interacting with microglia after traumatic brain injury, clarifying the regulatory role of migrasome generation and membrane fusion events in neuroinflammation [68]. Current research still faces technical and mechanistic challenges. Although DAOSLIMIT and 2pSAM have enhanced imaging depth and speed, the scattering effect of dense tissues still limits axial resolution, necessitating optimization through deep learning or nonlinear optical methods. On the functional level, the molecular composition, secretion regulatory network, and interaction mechanisms with the cytoskeleton of migrasomes have not been fully elucidated, urgently requiring integration of single-cell sequencing, spatial metabolomics, and gene editing technologies for systematic analysis.

Nonetheless, advances in purification techniques, the identification of reliable biomarkers, and the development of direct quantitative detection methods are gradually improving the conditions for in-depth migrasome research. While studies have identified key factors such as TSPAN4, cholesterol, sphingolipids, integrins, and *PIP5K1A* in migrasome formation, this also indicates that migrasomes may be regulated by upstream signals or other signaling cascades. Current research suggests that migrasomes contain a variety of substances and information, such as mRNA, proteins, chemokines, and growth factors. However, most of these studies have been limited to specific physiological conditions, such as embryonic development or tissue regeneration. The functions of migrasomes in different physiological and pathological contexts (e.g., immune responses, tumor microenvironments) remain insufficiently elucidated. Furthermore, regarding the fate of migrasomes, whether other pathways exist for their biological regulatory roles—beyond the release of contents into the extracellular environment or uptake by other cells—remains a topic for future investigation.

This review systematically discusses the biological formation, physiological functions, and pathological impacts of migrasomes, briefly exploring their potential applications in diagnosis and therapy. Given the numerous unknowns in the field of migrasomes, future research will need to continue striving to unveil the full extent of their roles and mechanisms.
